# The medically managed patient with severe symptomatic aortic stenosis in the TAVR era: Patient characteristics, reasons for medical management, and quality of shared decision making at heart valve treatment centers

**DOI:** 10.1371/journal.pone.0175926

**Published:** 2017-04-21

**Authors:** Kumar Dharmarajan, Jill Foster, Megan Coylewright, Philip Green, John P. Vavalle, Osman Faheem, Pei-Hsiu Huang, Amar Krishnaswamy, Vinod H. Thourani, Lisa A. McCoy, Tracy Y. Wang

**Affiliations:** 1 Section of Cardiovascular Medicine, Department of Internal Medicine, Yale University School of Medicine, New Haven, CT, United States of America; 2 Center for Outcomes Research and Evaluation, Yale-New Haven Hospital, New Haven, CT, United States of America; 3 American College of Cardiology, Washington, DC, United States of America; 4 Section of Cardiovascular Medicine, Dartmouth-Hitchcock Medical Center, Lebanon, NH, United States of America; 5 Division of Cardiology, Center for Interventional Vascular Therapy, Columbia University Medical Center, New York, NY, United States of America; 6 Division of Cardiology, Department of Medicine, University of North Carolina at Chapel Hill, Chapel Hill, NC, United States of America; 7 Prima Care Cardiology, Fall River, MA, United States of America; 8 Sutter Heart & Vascular Institute, Sacramento, CA, United States of America; 9 Department of Cardiovascular Medicine, Division of Interventional Cardiology, Heart and Vascular Institute, Cleveland Clinic, Cleveland, OH, United States of America; 10 Division of Cardiothoracic Surgery, Emory University School of Medicine, Atlanta, GA, United States of America; 11 Duke Clinical Research Institute, Durham, NC, United States of America; Universita degli Studi di Roma La Sapienza, ITALY

## Abstract

**Background:**

Little is known about patients with severe symptomatic aortic stenosis (AS) who receive medical management despite evaluation at a heart valve treatment center.

**Objective:**

We identified patient characteristics associated with medical management, physician-reported reasons for selecting medical management, and patients’ perceptions of their involvement and satisfaction with treatment selection.

**Methods and results:**

Of 454 patients evaluated for AS at 9 established heart valve treatment centers from December 12, 2013 to August 19, 2014, we included 407 with severe symptomatic AS. Information was collected using medical record review and survey of patients and treating physicians. Of 407 patients, 212 received transcatheter aortic valve replacement (TAVR), 124 received surgical aortic valve replacement (SAVR), and 71 received medical management (no SAVR/TAVR). Thirty-day predicted mortality was higher in patients receiving TAVR (8.7%) or medical management (9.8%) compared with SAVR (3.4%) (*P<*0.001). Physician-reported reasons for medical management included patient preference (31.0%), medical futility (19.7%), inoperability/anatomic infeasibility (11.3%), and inadequate vascular access (8.5%). Compared with patients receiving AVR, medically managed patients were less likely to report that they received enough information about the pros and cons of treatment options (*P* = 0.03), that their physicians involved them in treatment decisions (*P*<0.001), and that final decisions were the right ones (*P*<0.001).

**Conclusions:**

Patient preference was the most common physician-reported reason for selecting non-invasive AS management, yet patients not undergoing AVR after valve center evaluation reported being less likely to receive sufficient education about treatment options and more likely to feel uncertain about final treatment decisions. Greater attention to shared decision making may improve the experience of care for this vulnerable group of patients.

## Introduction

In the current era of transcatheter aortic valve replacement (TAVR), older patients receiving either TAVR or surgical aortic valve replacement (SAVR) for severe symptomatic aortic stenosis (AS) have been well characterized in terms of their risk profiles and outcomes. Pivotal clinical trials have described patients with severe, inoperable AS and their outcomes from TAVR,[1, 2] as well as patients at both high and intermediate surgical risk and their outcomes from both TAVR and SAVR.[3–6] Results from published studies have been supplemented by data from large registries[7–13] that have identified risk profiles, treatment approaches, and outcomes associated with aortic valve replacement (AVR) in real-world settings.

In contrast, little is known about patients with severe symptomatic AS who do not undergo AVR despite referral to a heart valve treatment center in the current era. Prior research on “medically managed” patients with AS has focused on describing the natural history of AS,[14–18] calculating the proportion of patients never referred for valve replacement,[19–24] and understanding reasons for non-referral.[21–24] Much less is known about the characteristics of patients receiving medical management despite referral for AVR, physicians’ rationales for these decisions,[25] and patients’ experience with care. In particular, we do not know if patients receiving medical management believe they have received sufficient education to inform decisions about AVR or had sufficient opportunities to articulate and incorporate their personal values and preferences into their care plans, all of which are core elements of shared decision making. We also do not know if these patients feel comfortable with medical management as a final treatment decision. More information about patients who receive medical management for severe symptomatic AS after valve center evaluation may identify multiple opportunities to improve their care at valve treatment centers, such as with improved education about treatment options, increased focus on effective implementation of shared decision making, and more optimized utilization of palliative care services.

In pursuit of this information, we studied contemporary data from the American College of Cardiology’s (ACC’s) educational and quality improvement initiative *Championing Care for the Patient with Aortic Stenosis*. One component of the initiative was to characterize the current context for AS care in the United States by surveying patients and their physicians at nine well-established heart valve treatment centers. We hypothesized that this data would demonstrate that patients receiving medical management after referral for AVR would have higher predicted mortality risk with surgery than patients receiving TAVR or SAVR and would be more likely to report deficiencies in core elements of shared decision making than patients receiving AVR. We also hypothesized that the most common reasons reported by physicians for selection of medical management would include medical futility and anatomic contraindications to AVR rather than patient preference.

## Methods

### Study population

Study patients were drawn from nine established heart valve treatment centers in the United States that were participating in the ACC’s initiative *Championing Care for the Patient with Aortic Stenosis*. The mission of this initiative was to empower members of the heart valve team to appropriately assess, refer, and provide timely patient-centered interventions for older patients with severe symptomatic AS. One component of the initiative involved characterizing the contemporary context of care for AS using a set of surveys developed to gather information from (1) patient participants who had been referred to, evaluated, and treated at participating valve centers, (2) participants’ medical records, and (3) participants’ consulting valve center physicians. Participating valve centers all met the following pre-specified criteria: (1) multidisciplinary heart valve team in place for at least six months; (2) minimum of six TAVR procedures completed each month; (3) participation in the Society of Thoracic Surgeons (STS)/ACC Transcatheter Valve Therapy (TVT) registry; and (4) prior or current participation in a TAVR clinical trial.

Each valve center was asked to recruit at least 60 patients referred for AS evaluation who subsequently received TAVR, SAVR, or medical management. Those patients who received medical management may have undergone balloon aortic valvuloplasty as a palliative procedure. To be eligible for participation, patients had to meet the following criteria: (1) age 19 years or older; (2) referral to a participating valve treatment center for evaluation and treatment of AS; (3) completion of TAVR, completion of SAVR, or decision made to receive medical management in the 18 months prior to study initiation; and (4) ability of the patient or his or her caregiver designee to complete all survey questions online, by paper-based instrument, or by verbal responses in person or by phone. Patients received no financial incentive for participation. Data collection occurred between December 12, 2013 and August 19, 2014.

### Data elements

A data coordinator at each valve center abstracted the medical record for each participating patient using a standardized form that was matched to the treatment strategy (TAVR, SAVR, or medical management). This form collected demographic information, past health history, clinical status at the time of evaluation, and aortic valve measurements obtained on echocardiography and/or cardiac catheterization. Data elements were modeled on the abstraction form used by the STS/ACC TVT registry.

For patients receiving medical management, the coordinator also reviewed the medical record and met with each patient’s consulting valve center physician to determine the reasons why medical management was chosen. Potential reasons included (1) patient preference; (2) medical futility due to a life expectancy of less than 12 months due to non-cardiac conditions, as determined by the evaluating physician; (3) acute myocardial infarction in the past 30 days; (4) contraindication to anti-thrombotic agents; (5) contraindication to TAVR due to insufficient vascular access or anatomic infeasibility of aortic annulus; and (6) other. The perceived timeliness of the patient’s referral to the valve treatment center (too early, early, timely, late, too late) was also determined based on review of the medical record and assessment by the valve center physician.

All participating patients also responded to a survey that assessed their experience at the valve treatment center. The survey included items that assessed patients’ (1) satisfaction with their initial appointment and diagnostic tests (e.g., laboratory testing, echocardiogram, cardiac catheterization); (2) belief that they received the necessary information to make decisions around valve replacement; (3) belief that they were given the chance by valve center physicians to articulate and incorporate their personal values and preferences into their care plan; and (4) belief that the final decision about their treatment for AS was the right one. Responses were collected using a Likert-type scale with the following choices: strongly disagree, disagree, neither agree nor disagree, agree, strongly agree, and don’t know or not applicable.

Data coordinators submitted the data for each patient into a secure, HIPAA-compliant, online form and database that was managed by the ACC. The online form was programmed to minimize missing data. Quality checks were performed regularly to identify and resolve all missing and erroneous data.

### Categorization of patients by treatment group

All patients were categorized as having received TAVR, SAVR, or medical management. Patients were excluded from our analysis if any of the following were present: (1) the treating physician characterized the timeliness of the consultation as too early; (2) the patient was asymptomatic, had mild or moderate AS, or had symptoms primarily from a non-cardiac condition; or (3) no reason besides “other” was given for the decision to pursue medical management and the patient had not previously received balloon aortic valvuloplasty, calculated aortic valve area was greater than 1 cm^2^, and mean aortic valve pressure gradient was less than 40 mm Hg.

### Study outcomes

We examined the following outcomes among patients with severe symptomatic AS receiving TAVR, SAVR, or medical management: (1) medical history and complexity, including STS-predicted 30-day mortality rate; (2) physician-reported reasons for medical management (if appropriate); and (3) patient-reported beliefs as to whether they had received sufficient education to inform decisions about AVR and sufficient opportunities to articulate and incorporate their personal values and preferences into their care plans.

### Statistical analysis

We calculated summary statistics for categorical variables using frequencies and percentages. We calculated summary statistics for continuous variables using means with standard deviations and medians with interquartile ranges. To compare differences in statistics between treatment groups, we used Wilcoxon rank-sum and Kruskal-Wallis tests for continuous variables and chi-square tests for categorical variables. When examining differences in patient characteristics by treatment strategy, we used 3-way testing to compare TAVR, SAVR, and medical management groups because of anticipated differences in risk profiles between patients receiving TAVR and SAVR. When examining differences in shared decision making by treatment strategy, we used 2-way testing to compare the combined TAVR/SAVR group with the medical management group because our intent was to understand differences in patient experience by the decision to undergo or not undergo definitive valve replacement. All statistical tests were 2-sided, and statistical significance was defined by a *P* value of less than 0.05. Analyses were conducted using SAS version 9.4 (Cary, NC) at the Duke Clinical Research Institute.

The ACC obtained Institutional Review Board approval for this study from Chesapeake IRB. Each participating valve treatment center (Cedars-Sinai, Cleveland Clinic, Duke University, Emory Healthcare, Mayo Clinic, New York Presbyterian Hospital of Columbia University Medical Center, the Heart Hospital at Baylor Plano, Saint Vincent Medical Group in Indianapolis, and Yale University) obtained Institutional Review Board approval for this study from its own institutional IRB. Duke University obtained a second Institutional Review Board approval from its own institutional IRB to support its role as the data analysis center for this study.

## Results

This study included 454 patients evaluated for severe symptomatic AS from nine heart valve treatment centers (enrollment numbers by center ranged from 34 to 61). Of 454 enrolled patients, 212 (46.7%) underwent TAVR, 124 (27.3%) SAVR, and 118 (26.0%) medical management. Of the 118 patients undergoing medical management, 47 (39.8%) were not felt to have severe symptomatic AS and were therefore excluded from subsequent analyses. Specifically, twenty patients were excluded because the treating physician characterized the consultation as too early. An additional 20 were excluded because the patient was described as asymptomatic, had mild or moderate AS, or had symptoms primarily from a non-cardiac condition. Seven patients were excluded because no reason except “other” was given for the decision to pursue medical management, the patient had not previously received balloon aortic valvuloplasty, calculated aortic valve area was greater than 1 cm^2^, and mean aortic valve pressure gradient was less than 40 mm Hg.

Characteristics of patients with severe symptomatic AS in TAVR, SAVR and medical management cohorts are shown in Table 1. Patients receiving medical management were more likely than those who received SAVR or TAVR to have class IV heart failure (*P =* 0.003) and be on home oxygen (*P =* 0.004). Patients receiving TAVR were more likely to have had previous coronary artery bypass procedures (*P<*0.001). Mean left ventricular ejection fraction, mean aortic valve pressure gradient, and mean aortic valve area were no different among treatment groups. The STS-predicted 30-day mortality rate was higher in patients undergoing TAVR or medical management compared to patients undergoing SAVR (*P<*0.001).

**Table 1:** Characteristics of patients receiving transcatheter aortic valve replacement, surgical aortic valve replacement, and medical management for severe symptomatic aortic stenosis.

Patient Characteristic	TAVR Cohort (N = 212)	SAVR Cohort (N = 124)	Medical Management Cohort (N = 71)	*P* Value
**Demographics**				
Mean age in years (SD)	80.7 (8.6)	73.2 (9.5)	82.6 (8.6)	<0.001
Male gender (%)	57.6	65.3	47.9	0.06
White race (%)	96.7	96.0	93.0	0.39
Black race (%)	2.8	2.4	4.2	0.77
**Cardiac history**				
Prior MI (%)	21.2	8.9	19.7	0.01
Prior PCI (%)	32.6	19.4	18.3	0.008
Prior CABG %)	40.6	6.5	22.5	<0.001
Prior aortic valve procedure (%)	10.9	2.4	7.0	0.02
NYHA class 3 (%)	50.0	31.5	38.0	0.003
NYHA class 4 (%)	30.2	17.7	39.4	0.003
Mean LVEF, in % (SD)	53.9 (12.5)	56.2 (10.7)	51.5 (15.3)	0.20
Mean AV pressure gradient in mm Hg (SD)	45.4 (13.0)	44.6 (13.9)	41.2 (13.8)	0.12
Mean AV area in cm^2^ (SD)	0.7 (0.2)	0.8 (0.2)	0.7 (0.2)	0.07
**Non-cardiac history**				
Prior stroke (%)	10.8	5.7	14.1	0.13
Peripheral arterial disease (%)	44.3	17.7	31.0	<0.001
Diabetes mellitus (%)	39.2	33.1	39.4	0.50
End-stage renal disease (%)	2.4	2.4	7.0	0.13
Moderate or severe chronic lung disease (%)	32.6	12.9	31.0	<0.001
On home oxygen (%)	15.1	4.8	19.7	0.004
Mean serum creatinine in mg/dL (SD)	1.3 (0.7)	1.2 (1.2)	1.6 (1.6)	0.43
**Mean STS 30-day predicted mortality rate (%) (SD)**	8.7 (4.5)	3.4 (2.4)	9.8 (6.4)	<0.001

AV, aortic valve; CABG, coronary artery bypass grafting; LVEF, left ventricular ejection fraction; MI, myocardial infarction; NYHA, New York Heart Association; PCI, percutaneous coronary intervention; SAVR, surgical aortic valve replacement; SD, standard deviation; STS, Society of Thoracic Surgeons; TAVR, transcatheter aortic valve replacement.

**Fig 1:**
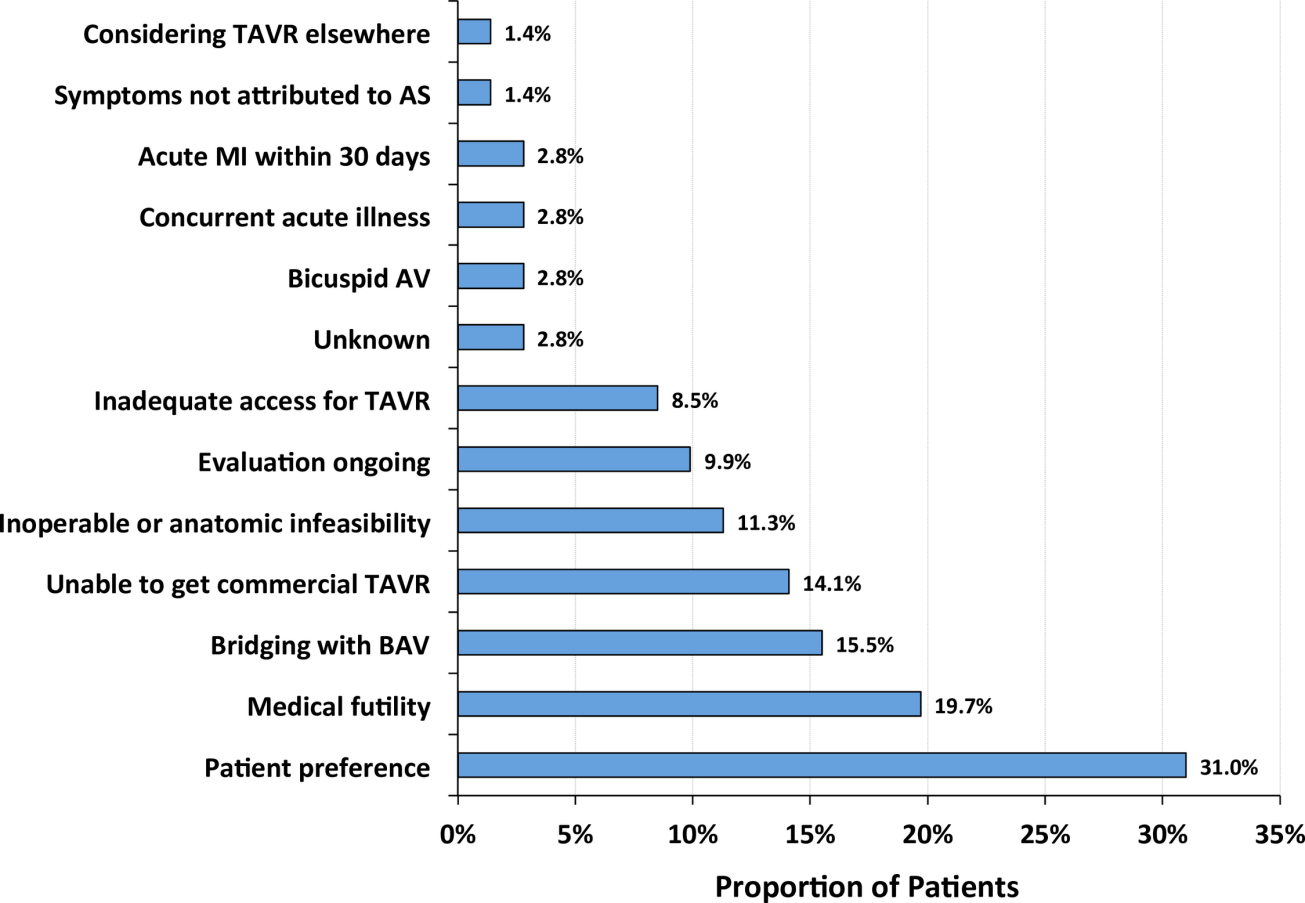
Reasons for medical management among patients with severe symptomatic aortic stenosis (AS). The total number of articulated reasons was divided by the number of included patients (n = 71), as patients could have more than one reason for medical management. AV indicates aortic valve; BAV, balloon aortic valvuloplasty; MI, myocardial infarction; and TAVR, transcatheter aortic valve replacement.

Physician-reported reasons for selecting medical management for patients with severe symptomatic AS are presented in Fig 1. The most common reason was patient preference, which was present in 31% of patients who received medical management after valve center evaluation. Almost 20% of patients did not receive valve replacement because of physicians’ concerns of medical futility, and approximately 11% and 9% of patients did not receive TAVR or SAVR because of inoperability/anatomic infeasibility or inadequate access for TAVR, respectively. Notably, 14% of medically managed patients declined surgery in the hope that access to TAVR would broaden in the future.

**Fig 2:**
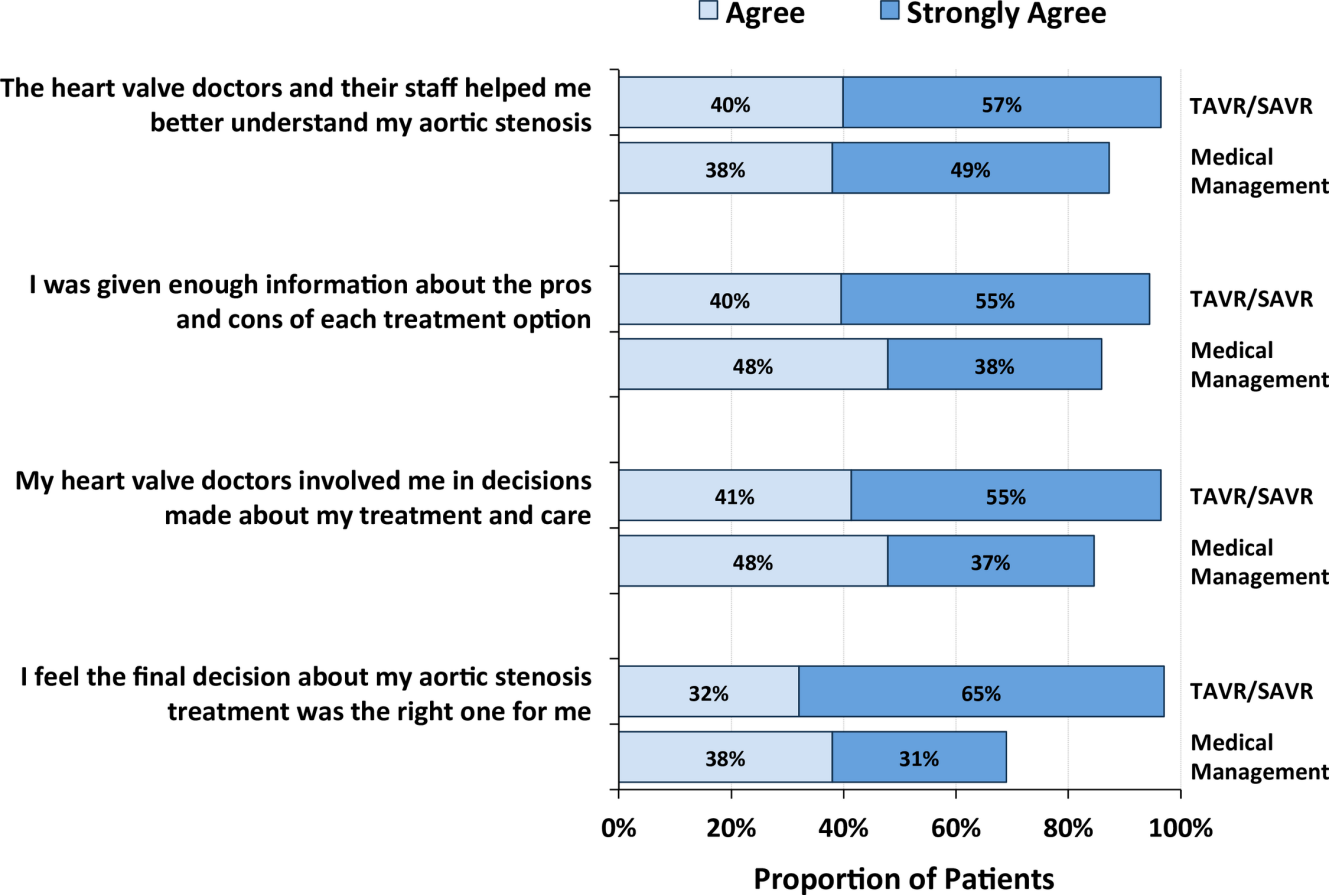
Quality of knowledge transfer, patient engagement, and shared decision making by aortic valve treatment strategy. Bar charts compare responses of “agree” or “strongly agree” to study questions between participants who did and did not receive aortic valve replacement. AS, aortic stenosis; SAVR, surgical aortic valve replacement; TAVR, transcatheter aortic valve replacement.

Data from patients’ self-reports on the presence of elements related to shared decision making during valve center evaluation are presented in S1 Table. While the majority of patients had favorable experiences during valve center evaluation, those receiving medical management compared with AVR were less likely to believe that they had received the necessary information to make decisions regarding valve replacement (Fig 2).

Medically managed patients were less likely to believe that heart valve physicians and staff helped them better understand their AS (*P =* 0.01), less likely to believe that they were given enough information about the pros and cons of each treatment option (*P =* 0.03), and less likely to believe that their heart valve physicians involved them in decisions around treatment (*P*<0.001). Patients receiving medical management were also less likely to believe that the final treatment decision was the right one for them (*P*<0.001). In particular, only 69% of patients receiving medical management agreed or strongly agreed that this treatment course was the right one for them, whereas over 95% of patients receiving TAVR or SAVR agreed or strongly agreed that the decision to pursue definitive valve replacement was the correct one.

## Discussion

This study is unique in characterizing patient-centered outcomes among patients with severe symptomatic AS who were evaluated at a heart valve treatment center, but did not receive either TAVR or SAVR. Our findings demonstrate that such patients have significant medical complexity and frequent medical and technical contraindications to valve replacement. While the majority of patients had favorable experiences during valve center evaluation, those receiving medical management were less likely to report receiving sufficient information to support the decision for medical management, less likely to feel that their heart valve doctors involved them in treatment decisions, and less likely to believe that the final decision was the right one for them. Overall, these results suggest a potential gap in care that may benefit from additional efforts to enhance communication between patients and physicians.

Interventional cardiologists and cardiac surgeons who are poised to deliver AVR may not always be skilled in shared decision making and alternative treatment modalities, including palliative care. To promote enhanced communication, valve centers may consider supplementing their current interactions with patients with (1) in-visit decision aids designed to increase collaborative discussions with providers; (2) educational material focused on medical management, such as tailored after-visit summaries that explain the rationale for not selecting AVR; and (3) video presentations that review potential risks of treatment that may result in further suffering. Such tools have been used to facilitate discussions around cardiopulmonary resuscitation.[26, 27] While it is true that disappointment in the final recommendation against AVR may influence some patients’ perceptions of their valve center experience, feelings of frustration and exclusion are real. Additional engagement and time discussing treatment alternatives and priorities may improve the degree to which patients feel that their goals, values, and preferences are sufficiently understood and acknowledged.[28] In addition, further attention to patients’ emotional responses may improve their satisfaction with communication.[29] These emotional reactions can be just as important to address as more cognitive responses.[29] While previous work has shown that patients’ emotional concerns are often unrecognized and unaddressed,[30, 31] intensive communication skills training using role playing in small groups can help physicians of multiple disciplines provide better emotional support to patients who receive bad news or a recommendation for palliative care.[32–35]

As more than one in three patients did not receive AVR due to anatomic contraindications or a high competing risk of death from other medical conditions, it is likely that many patients will benefit from palliative care consultation. Palliative care is a recommended component of care for medically managed symptomatic AS[36, 37] that can provide supportive resources to help patients understand the expected course of disease, recognize their concerns and fears, discuss spiritual and existential needs, and develop plans for symptom management as disease progresses.[38–40] Palliative care consultation can also help stimulate advanced care planning and goal setting in which patients “hope for the best but plan for the worst.”[38] Valve treatment centers may therefore consider developing internal palliative care resources to start preliminary discussions with all patients for whom a decision is made to forgo valve replacement [41] and may also consider developing relationships with outpatient palliative care providers who can provide more comprehensive services.

This study draws attention to the larger group of patients with advanced cardiovascular disease who do not undergo potentially life-saving or palliative procedures for which they have been evaluated. Cardiologists and cardiothoracic surgeons see many such individuals, including those with pronounced angina who are not candidates for complex revascularization procedures and patients with advanced heart failure who are not candidates for heart transplant or a ventricular assist device. Currently, no registries have been established to study the experiences of these patients during and after consultation. Patients with cardiovascular disease may be especially likely to experience suboptimal end of life care compared with persons with cancer and dementia.[42] Due to their significant patient volume, large referral centers are in an ideal position to advance our knowledge of these medically managed patients and can facilitate prospective observational studies and clinical trials to better characterize patient phenotypes, disease progression, and responses to various interventions to help improve important outcomes such as symptom burden, functional status, independence, and health-related quality of life.[43, 44]

Our results should be interpreted in the context of the following limitations. First, our study is unique in collecting patient perspectives on referral outcomes, but is based on a relatively small sample size drawn from nine leading heart valve treatment centers that may not provide care that is broadly generalizable. However, our finding of deficiencies in information transfer and core elements related to shared decision making at these established centers suggests that these gaps in care may be widespread. Second, our study population was not randomly selected. However, the population that was enrolled likely biased results to the null, as medically managed patients who were well enough to participate in our study should have been less likely to have high risk features and multiple contraindications to valve replacement. Third, we did not follow medically managed patients to define disease progression, as this was not our goal. Our intent was to describe the characteristics of patients with severe symptomatic AS at the time of valve center evaluation, understand the reasons why medical management is most often pursued, and characterize these patients’ experience of care around the decision to forgo valve replacement. As a result, we interviewed patients and their treating physicians to better understand complexities associated with evaluation and management. Fourth, survey results of medically managed patients on the quality of interactions during valve center evaluation may be disproportionally biased by dissatisfaction with the treatment decision.

In summary, we found that patients receiving medical management for severe symptomatic AS after referral to a heart valve treatment center are medically complex and frequently have contraindications to TAVR and SAVR. While physicians report patient preference as the most common reason to select medical management, we observed that patients receiving medical management are less likely to report receiving sufficient information to make decisions around valve replacement, less likely feel engaged by their heart valve physicians in treatment decisions, and less likely to believe that the final decision was the appropriate one. Additional investment in patient education and engagement, provider training, and palliative care services may help improve patients’ experience during and after valve center evaluation.

## Supporting information

S1 Table Quality of knowledge transfer, patient engagement, and shared decision making by aortic valve treatment strategy. (DOCX)

S1 File Raw study data and data dictionary. (XLSX)

S2 File Coded reasons for medical management. (XLSX)
