# Supplementary material for: The medically managed patient with severe symptomatic aortic stenosis in the TAVR era: Patient characteristics, reasons for medical management, and quality of shared decision making at heart valve treatment centers
Source: PLoS One. 2017 Apr 21;12(4):e0175926. doi: 10.1371/journal.pone.0175926 (PMC5400246; doi:10.1371/journal.pone.0175926)
Supplement: S1 Table — (DOCX) [file pone.0175926.s001.docx]

**S1 Table. Quality of Knowledge Transfer, Patient Engagement, and Shared Decision Making by Aortic Valve Treatment Strategy**

|  |  | TAVR/SAVR Cohorts (N=336) | | Medical Management Cohort (N=71) | | *P* Value |
| --- | --- | --- | --- | --- | --- | --- |
| Question Category and Questions | **Response** | **n** | **%** | **n** | **%** |  |
| *Knowledge transfer* |  |  |  |  |  |  |
| The heart valve doctors and their staff helped me better understand my AS. | Strongly Disagree | 1 | 0.3 | 0 | 0 | 0.01 |
|  |  |  |  |  |  |  |
|  | Disagree | 1 | 0.3 | 0 | 0 |  |
|  | Neither Agree nor Disagree | 8 | 2.4 | 9 | 12.7 |  |
|  | Agree | 134 | 39.9 | 27 | 38.0 |  |
|  | Strongly Agree | 190 | 56.6 | 35 | 49.3 |  |
|  | Don't Know or Not Applicable | 2 | 0.6 | 0 | 0 |  |
| Treatment options were explained to me in a way that was easy to understand. | Strongly Disagree | 1 | 0.3 | 0 | 0 | 0.01 |
|  | Disagree | 1 | 0.3 | 0 | 0 |  |
|  | Neither Agree nor Disagree | 5 | 1.5 | 2 | 2.82 |  |
|  | Agree | 136 | 40.5 | 41 | 57.8 |  |
|  | Strongly Agree | 190 | 56.5 | 25 | 35.2 |  |
|  | Don't Know or Not Applicable | 3 | 0.9 | 3 | 4.2 |  |
| Important questions I had were answered to my satisfaction. | Strongly Disagree | 1 | 0.3 | 0 | 0 | 0.73 |
|  | Disagree | 0 | 0 | 0 | 0 |  |
|  | Neither Agree nor Disagree | 8 | 2.4 | 3 | 4.2 |  |
|  | Agree | 132 | 39.3 | 30 | 42.2 |  |
|  | Strongly Agree | 195 | 58.0 | 38 | 53.5 |  |
|  | Don't Know or Not Applicable | 0 | 0 | 0 | 0 |  |
| I was given enough information about the pros and cons of each treatment option. | Strongly Disagree | 1 | 0.3 | 0 | 0 | 0.03 |
|  | Disagree | 0 | 0 | 0 | 0 |  |
|  | Neither Agree nor Disagree | 14 | 4.2 | 8 | 11.3 |  |
|  | Agree | 133 | 39.6 | 34 | 47.9 |  |
|  | Strongly Agree | 184 | 54.8 | 27 | 38.0 |  |
|  | Don't Know or Not Applicable | 4 | 1.2 | 2 | 2.8 |  |
| *Incorporation of values, preferences, patients, family members into decision making process* |  |  |  |  |  |  |
| The heart valve doctors listened to me and asked about the values and preferences that were important to me. | Strongly Disagree | 1 | 0.3 | 0 | 0 | 0.29 |
|  | Disagree | 0 | 0 | 0 | 0 |  |
|  | Neither Agree nor Disagree | 14 | 4.2 | 5 | 7.0 |  |
|  | Agree | 143 | 42.6 | 38 | 53.5 |  |
|  | Strongly Agree | 173 | 51.5 | 27 | 38.0 |  |
|  | Don't Know or Not Applicable | 5 | 1.5 | 1 | 1.4 |  |
| My heart valve doctors involved me in decisions made about my treatment and care. | Strongly Disagree | 1 | 0.3 | 0 | 0 | <0.001 |
|  | Disagree | 0 | 0 | 2 | 2.8 |  |
|  | Neither Agree nor Disagree | 7 | 2.1 | 8 | 11.3 |  |
|  | Agree | 139 | 41.4 | 34 | 47.9 |  |
|  | Strongly Agree | 185 | 55.1 | 26 | 36.6 |  |
|  | Don't Know or Not Applicable | 4 | 1.2 | 1 | 1.4 |  |
| My family and caregivers were able to join discussions about my condition and my treatment options, if desired. | Strongly Disagree | 1 | 0.3 | 0 | 0 | 0.89 |
|  | Disagree | 1 | 0.3 | 0 | 0 |  |
|  | Neither Agree nor Disagree | 8 | 2.4 | 3 | 4.2 |  |
|  | Agree | 117 | 34.8 | 26 | 36.6 |  |
|  | Strongly Agree | 207 | 61.6 | 42 | 59.2 |  |
|  | Don't Know or Not Applicable | 2 | 0.6 | 0 | 0 |  |
| *Overall patient satisfaction with decision* |  |  |  |  |  |  |
| I feel the final decision about my aortic stenosis treatment was the right one for me. | Strongly Disagree | 1 | 0.3 | 0 | 0 | <0.001 |
|  | Disagree | 0 | 0 | 0 | 0 |  |
|  | Neither Agree nor Disagree | 7 | 2.1 | 11 | 15.5 |  |
|  | Agree | 108 | 32.1 | 27 | 38.0 |  |
|  | Strongly Agree | 218 | 64.9 | 22 | 31.0 |  |
|  | Don't Know or Not Applicable | 2 | 0.6 | 11 | 15.5 |  |

AS, aortic stenosis; SAVR, surgical aortic valve replacement; TAVR, transcatheter aortic valve replacement
